# Ajuba Overexpression Promotes Breast Cancer Chemoresistance and Glucose Uptake through TAZ-GLUT3/Survivin Pathway

**DOI:** 10.1155/2022/3321409

**Published:** 2022-02-07

**Authors:** Xiang Li, Gexin Zhao, Xiaoyi Mi, Tonghong Xu, Xinmin Li, Bin Liu

**Affiliations:** ^1^Departments of Breast Surgery, Cancer Hospital of China Medical University, Liaoning Cancer Hospital and Institute, Shenyang, China; ^2^Samuel Oschin Comprehensive Cancer Institute, Cedars Sinai Medical Center, Los Angeles, CA, USA; ^3^Department of Pathology, College of Basic Medical Sciences and the First Affiliated Hospital, China Medical University, Shenyang, China; ^4^Departments of Medical Oncology, Cancer Hospital of China Medical University, Liaoning Cancer Hospital and Institute, Shenyang, China; ^5^Technology Center for Genomic & Bioinformatics, Department of Pathology and Laboratory Medicine, University of California, Los Angeles, USA

## Abstract

The LIM protein Ajuba has been implicated in the development of human cancers. To date, its expression pattern and biological significance in breast cancers (BC) have not been fully investigated. In the current study, we examined Ajuba protein levels in 93 invasive ductal carcinoma specimens by immunohistochemistry. The Ajuba expression level was elevated in breast cancer tissue compared with normal tissue. Ajuba overexpression is correlated with advanced tumor-node-metastasis (TNM) stage, positive node status, and adverse patient outcomes. The Ajuba protein level was also higher in BC cell lines compared to normal breast epithelial cell line MCF-10A. Ectopically expressed Ajuba in MCF-7 cells stimulated in vitro and in vivo cell growth, invasion, cell cycle progression, and decreased paclitaxel-induced apoptosis. RNA-sequencing (RNA-seq) followed by gene set enrichment analysis (GSEA) analysis showed that Ajuba overexpression regulated the Hippo signaling pathway. Ajuba overexpression also increased glucose uptake and increased expression of TAZ, GLUT3, and Survivin. TAZ knockdown abolished the role of Ajuba on GLUT3 and Survivin induction. The ChIP assay showed that TEAD4, a major TAZ binding transcription factor, could bind to the GLUT3 and Survivin promoter regions. In conclusion, our data demonstrated that elevated Ajuba expression is correlated with poor BC prognosis and regulated malignant behavior through TAZ-GLUT3/Survivin signaling in BC cells.

## 1. Introduction

Breast cancer (BC) is one of the most commonly diagnosed cancers and the second leading cause of cancer-related deaths in women [1–4]. In the past decades, the development of novel therapeutic treatments has reduced mortality and improved the survival of breast cancer [5]. However, due to chemotherapy resistance and lack of efficient treatment for highly aggressive subtypes such as TNBC, the prognosis of advanced breast cancer remains poor. Therefore, there is a compelling need for identifying novel biomarkers and therapeutic targets to improve the overall outcome of breast cancer patients [6–9].

Ajuba is a LIM family member (Ajuba, LIMD1, and WTIP) [10], which is characterized by tandem LIM protein in the C-termini. As an adaptor protein that could shuttle between the cytoplasm and the nucleus, Ajuba interacts with various proteins to form various complexes involving multiple signaling pathways [11–13]. Recently, several studies imply that Ajuba interacts with the Hippo pathway's core components, including LATS and WW45, and acts as a negative regulator of Hippo signaling [10, 14].

It has been reported that Ajuba is involved in the development of various human cancers. Ajuba expression is increased in cervical cancer [15], esophageal squamous cell carcinoma (ESCC) [16], colorectal cancer (CRC) [17, 18], gastric cancer [19], and pancreatic cancer [20]. Ajuba has also been implicated in a variety of oncogenic processes. Ajuba promotes cell proliferation in CRC and pancreatic cancer cells [17, 21]. Ajuba promotes ESCC cell invasion by activating ERK1/2 [16]. Ajuba augments tumor metastasis [18] and inhibits apoptosis [17]. Mutations of Ajuba regulate drug sensitivity of head and neck squamous cell carcinoma (HNSCC) [22]. Although growing evidence suggests that Ajuba acts as an oncogene to promote tumorigenesis, several studies reported Ajuba could function as a tumor suppressor [23, 24]. To date, the expression pattern and biological functions of Ajuba in human breast cancers have not been fully elucidated.

## 2. Materials and Methods

### 2.1. Specimens

The current protocol was reviewed and approved by the Institutional Review Board, Cancer Hospital of China Medical University. This study was carried out following the Declaration of Helsinki principle. Written informed consent was obtained from patients. Breast cancer specimen paraffin blocks were from the Pathology Department in the First Affiliated Hospital of China Medical University, containing specimens no longer required to be maintained.

### 2.2. Immunohistochemistry

Immunohistochemistry was performed according to protocols reported previously [25]. In brief, 4 *μ*m paraffin sections were deparaffinized using xylene and treated with ethanol. Peroxidase blocking was performed using H_2_O_2_ solution (concentration: 3% *v*/*v*). Antigen retrieval was performed using citrate buffer. After blocking with ready-to-serum and incubation with Ajuba primary antibody (1 : 100, Sigma, USA), the section was treated with the Elivision plus Kit from Maixin (Fuzhou, China) and immunostaining was developed using the DAB kit. Counterstaining was performed using hematoxylin.

Ajuba staining was scored according to the immunoreactive score (IRS), which was reported previously [26, 27]. The intensity of staining was scored as 0 negative, 1 moderate, and 2 strong, and the percentage of positive expression was categorized as 1 (<25%), 2 (25-50%), 3 (50-75%), and 4 (75-100%). The final scores were obtained by multiplying the intensity score with the percentage of positive expression. The section with a score < 4 was considered low expression. The section with a score ≥ 4 was considered overexpression.

### 2.3. Cell Culture

Breast cancer cell lines, including MDA-MB-453, MDA-MB-468, BT474, BT549, T47D, MCF7, SK-BR-3, and the human normal breast cell line (MCF-10A), were obtained from American Type Culture Collection. BC cells were cultured using the RPMI-1640 supplied with 10% (*v*/*v*) fetal bovine serum (FBS). MCF-10A cells were cultured in DMEM/F12 medium supplemented with 10% (*v*/*v*) fetal bovine serum (FBS), 20 ng/ml EGF, 0.5 mg/ml hydrocortisone, 10 *μ*g/ml insulin, and 100 ng/ml cholera toxin.

The Ajuba plasmid and the corresponding negative pCMV6 empty vector were from Origene company and transfected into cells using Lipofectamine 3000. Ajuba specific siRNA was from Dharmacon and transfected using the Dharmafect1 reagent. All transfection procedures were conducted following the manufacturer's protocol.

### 2.4. Western Blot

Protein was extracted from cells using RIPA buffer. The protein was denatured in loading buffer at 100°C for 5 minutes. 40 *μ*g protein was separated using SDS-PAGE and transferred to the PVDF membrane. The PVDF membrane was incubated with the following antibodies including Ajuba (HPA006171) (1 : 800, Sigma, USA), Survivin (#2808), TAZ (#83669), GAPDH (#5174) (1 : 1000, Cell Signal Technology, USA), and GLUT3 (ab191071) (1 : 1000, Abcam, USA). The membranes were then washed with TBS-T and incubated with peroxidase-conjugated secondary antibody at room temperature for 2 hours. Finally, the protein was visualized using an ECL kit. Relative protein levels were quantified using ImageJ.

### 2.5. Real-Time PCR

Total RNA was extracted using RNAiso (TaKaRa, Dalian, China) and reverse transcribed into cDNA using the Prime Script RT MasterMix Kit (TaKaRa). Real-time PCR was conducted using SYBR MasterMix (Thermo) according to the manufacturer's instructions. mRNA expression of target genes was normalized to GAPDH using the 2^-*ΔΔ*ct^ method. The primer sequences were as follows: Ajuba for 5′-GATGCGGGAGCCAGAGG-3′, rev 5′-CACAAGAGCAGCAAACAAAGC-3′; Survivin for 5′-ACCGCATCTCTACATTCAAG-3′, rev 5′-CAAGTCTGGCTCGTTCTC-3′; GLUT3 for 5′-CCTTTGGCACTCTCAACCAGC-3′, rev 5′-AACCCAGTAGCAGCGGCCAT-3′; and GADPH for 5′-GAAATCCCATCACCATCTTCCAG-3′, rev 5′-GAGCCCCAGCCTTCTCCAT-3′.

### 2.6. RNA-Sequencing

The RNA-sequencing experiments were carried out by Novogene corporation (Beijing, China). The libraries were sequenced on an Illumina NovaSeq6000 platform. The sequence data was subjected to standard quality control (QC). Gene set enrichment analysis (GSEA) is performed using the software downloaded from the GSEA website (http://software.broadinstitute.org).

### 2.7. CCK-8 and Colony Formation Assays

CCK-8 and colony formation assays were performed according to protocols reported previously [25, 28]. For CCK-8, cells were firstly plated into 96-well plates (3000 cells/well). Ten *μ*l of the CCK-8 reagent (Cell Counting Kit-8; Dojindo, Kumamoto, Japan) was added into each well. After incubation for 2 hours, absorbance was examined using a microplate reader (wavelength: 450 nm). For colony formation capacity, cells were seeded into 6 cm plates (concentration: 1000 cells/plate) and cultured for about two weeks. The cells were then stained using Giemsa and counted under a microscope. The experiment was repeated three times.

### 2.8. Transwell Invasion Assay

The invasion assay was performed using 24-well transwell chambers (costar, 8 *μ*m pore), and the chambers were coated with matrigel (BD bioscience) at 37°C for 4 hours. Cell mixture with no FBS was plated in the top chamber. Medium supplied with 10% (*v*/*v*) FBS was placed in the lower chamber. Subsequently, the plate was incubated at 37°C for 18-24 h. Invading cells at the bottom of the transwell were stained using 0.1% hematoxylin. The invading cell number was counted using a microscope.

### 2.9. Cell Cycle Transition and Apoptosis

Cell cycle transition and cell apoptosis rates were investigated after cells were modified. The modified cells were treated with 0.5% trypsin and washed with PBS butter. For the cell cycle assay, cells were fixed in 1% paraformaldehyde and stained with 5 mg/ml propidium iodide. The apoptosis assay was performed using the BD Annexin V/FITC kit (BD, USA) and detected using a flow cytometer.

### 2.10. Chromatin Immunoprecipitation (ChIP) Assay

The chromatin immunoprecipitation (ChIP) assay was performed using the Magna ChIP A/G Assay Kit (Millipore, CA, USA). Briefly, cells were crosslinked with 37% formaldehyde. The DNA/protein complexes were treated using TEAD4 and IgG (Cell signaling technology) antibodies and protein A/G magnetic beads. The precipitated chromatin complexes were purified and decrosslinked at 62°C for 2 h. The precipitated DNA fragments were quantified using PCR analysis. The primers for ChIP were listed as follows: SLC2A3 position1 forward, 5′ GTAATCTAGTTTTCTCGGGTCCAG3′; SLC2A3 position1 reverse, 5′ TTTCCCAGTGGTGAATTGGAG3′; SLC2A3 position2 forward, 5′CCACTGTGCCCAGGTCAAC 3′; SLC2A3 position2 reverse, 5′AGGGAAACCCCATCTCCAA 3′; BIRC5 position1 forward, 5′AAATCAGAGCTGGGGTCCAA3′; BIRC5 position1 reverse, 5′TGAAATCCCTGAGAAGCAGAGTG3′; BIRC5 position2 forward, 5′CTCTCACAGCCTTCTCTTGTCA 3′; and BIRC5 position2 reverse, 5′ CACCCCGAGGTACGATCAGT 3′.

### 2.11. Glucose Uptake

The glucose uptake assay was performed as previously reported [19, 25]. Briefly, cells were washed and resuspended in PBS with 2% FBS (FBS/PBS) and treated with the 2-NBDG (Thermo Fisher Scientific, USA) at a final concentration of 10 *μ*M for 30 minutes. Then, these cells were washed and resuspended in 300 *μ*l 2% FBS/PBS. The fluorescence intensity was measured using a flow cytometer.

### 2.12. Nude Mouse Xenograft

BALB/c athymic nude mice (4 weeks old) were purchased from Shanghai Slac Laboratory Animals (Shanghai, China). All animal experiments and procedures conformed to the institutional animal care guidelines. A xenograft model was established by subcutaneous right armpit injections of stable cell lines (5 million cells). Tumor size was measured each 7 days. Animals were sacrificed, and xenograft tumors were removed after 6 weeks.

### 2.13. Statistical Analysis

Statistical analysis was carried out using the software package SPSS, version 16.0 (SPSS, Chicago, IL). The *χ*^2^ test was used to analyze the possible associations between Ajuba status and clinical factors. Difference in patient survival was analyzed using Kaplan-Meier curves with Log-rank tests. Student's *t*-test was used to assess the difference in other experiments. *p* < 0.05 was regarded statistically significant.

## 3. Results

### 3.1. Ajuba Is Overexpressed in BC and Correlates with Clinicopathological Factors

**Figure 1:**
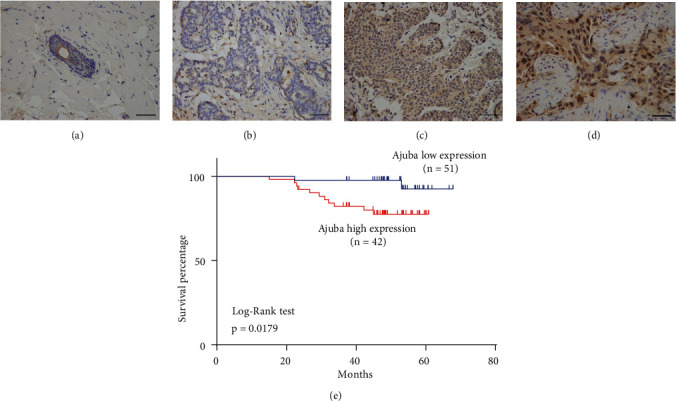
Expression pattern of Ajuba in breast cancer specimen. (a) Ajuba expression is negative in normal breast tissue. (b) Negative Ajuba expression in a case of invasive ductal carcinoma. (c) Moderate cytoplasmic Ajuba expression in a case of invasive ductal carcinoma. (d) Strong cytoplasmic and nuclear Ajuba expression in a case of invasive ductal carcinoma. (e) Kaplan-Meier curve and Log-rank test showed that high Ajuba status is associated with poor patient survival (*p* = 0.0179) (scale bar indicates 50 *μ*m).

Ajuba protein levels were examined in 93 invasive ductal carcinoma (IDC) samples and 15 normal breast tissue samples by immunohistochemistry (IHC). Normal tissues showed negative/weak Ajuba staining (Figure 1(a)). In 93 cases examined, 51 (54.8%) cases showed high Ajuba expression (Figures 1(b)–1(d)). Ajuba was located in the cytoplasm with nuclear staining in some cases. High Ajuba expression is positively associated with advanced TNM stage (*p* = 0.0009) and lymph node metastasis (*p* = 0.0007) (Table 1). Importantly, Ajuba expression in triple-negative breast carcinoma (TNBC) was higher than that in non-triple-negative breast carcinoma (*p* = 0.0196) (Table 1). Additionally, we found a significant association between Ajuba status and poor patient prognosis, which was analyzed using the Log-rank test (*p* = 0.0179, Figure 1(e)).

**Figure 2:**
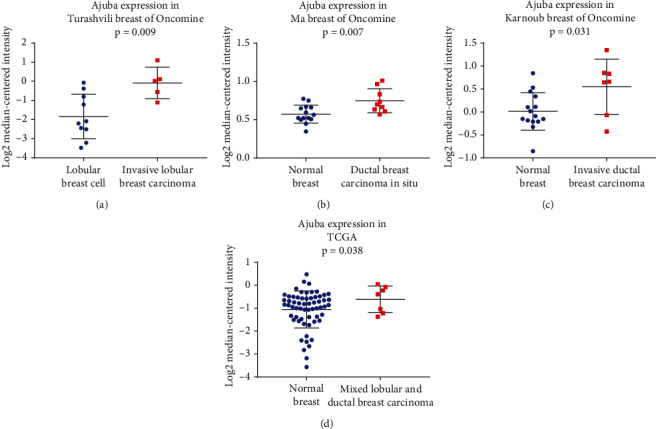
Analysis of Oncomine and TCGA data. (a) Analysis of Turashvili Oncomine data indicated that Ajuba mRNA was upregulated in invasive lobular breast carcinoma compared with normal lobular breast cells. (b) Ma breast dataset of Oncomine indicated that Ajuba was higher in ductal breast carcinoma in situ compared with normal breast tissue. (c) Karnoub breast dataset indicated that Ajuba level in invasive ductal breast carcinoma was high than that in normal breast. (d) TCGA dataset showed that Ajuba mRNA was higher in mixed lobular and ductal carcinoma compared to normal breast tissues. ^∗^*p* < 0.05.

Oncomine data was also analyzed. The Turashvili dataset of Oncomine indicated that Ajuba mRNA was higher in invasive lobular breast carcinoma than normal lobular breast cells (*p* = 0.009, Figure 2(a)). The Ma breast dataset showed that Ajuba expression was higher in ductal breast carcinoma in situ (*p* = 0.007, Figure 2(b)). The Karnoub breast dataset showed that Ajuba in invasive ductal breast carcinoma was higher compared with that of normal breasts (*p* = 0.031, Figure 2(c)). TCGA data also indicated that Ajuba mRNA was higher in mixed lobular and ductal carcinoma than normal breast tissues (*p* = 0.038, Figure 2(d)).

### 3.2. Ajuba Regulates Proliferation and Invasion in Breast Cancer Cells

**Figure 3:**
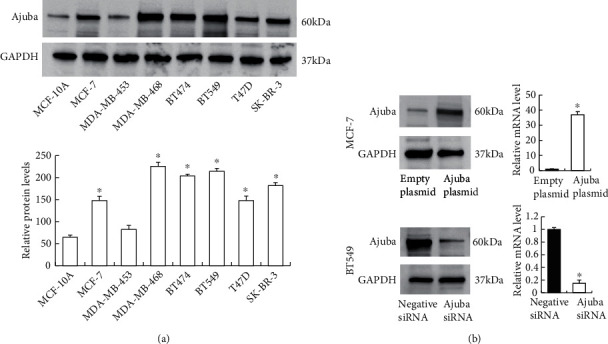
Ajuba expression and transfection efficiency in breast cancer cell lines. (a) Western blot was performed in a panel of breast cancer cell lines. The results showed that Ajuba protein was lower in MCF-10A cell line. Ajuba expression was higher in breast cancer cell lines (MCF-7, MDA-MB-468, BT474, BT549, T47D, and SK-BR-3), especially in triple-negative cell lines MDA-MB-468 and BT549. Relative protein levels were quantified using ImageJ. ^∗^*p* < 0.05 compared with MCF-10A. (b) Transfection and siRNA knockdown efficiencies were confirmed by western blot and RT-qPCR in MCF-7 and BT549, respectively. ^∗^*p* < 0.05.

Western blot was used to determine protein levels of Ajuba in normal breast epithelial cell line MCF-10A and a panel of cancer cell lines, including triple-negative cell lines MDA-MB-468 and BT549 and luminal cell lines MCF-7, MDA-MB-453, BT474, T47D, and SK-BR3. Relative protein levels were quantified using ImageJ. As shown in the Figure 3(a) histogram, the Ajuba protein level was relatively higher in BC cell lines (including MCF-7, BT474, T47D, SK-BR3, MDA-MB-468, and BT549) than normal breast MCF-10A cell line (*p* < 0.05). Histogram indicated that Ajuba protein levels showed the highest levels in triple-negative cell lines MDA-MB-468 and BT549 (Figure 3(a)).

Ajuba overexpression and knockdown were performed in MCF-7 and BT549 cell lines, respectively. Western blot showed successful transfection of Ajuba plasmid into MCF-7 cells. Ajuba knockdown significantly downregulated the endogenous Ajuba level in the BT549 cell line. Transfection efficiency was also confirmed by RT-qPCR (Figure 3(b)).

**Figure 4:**
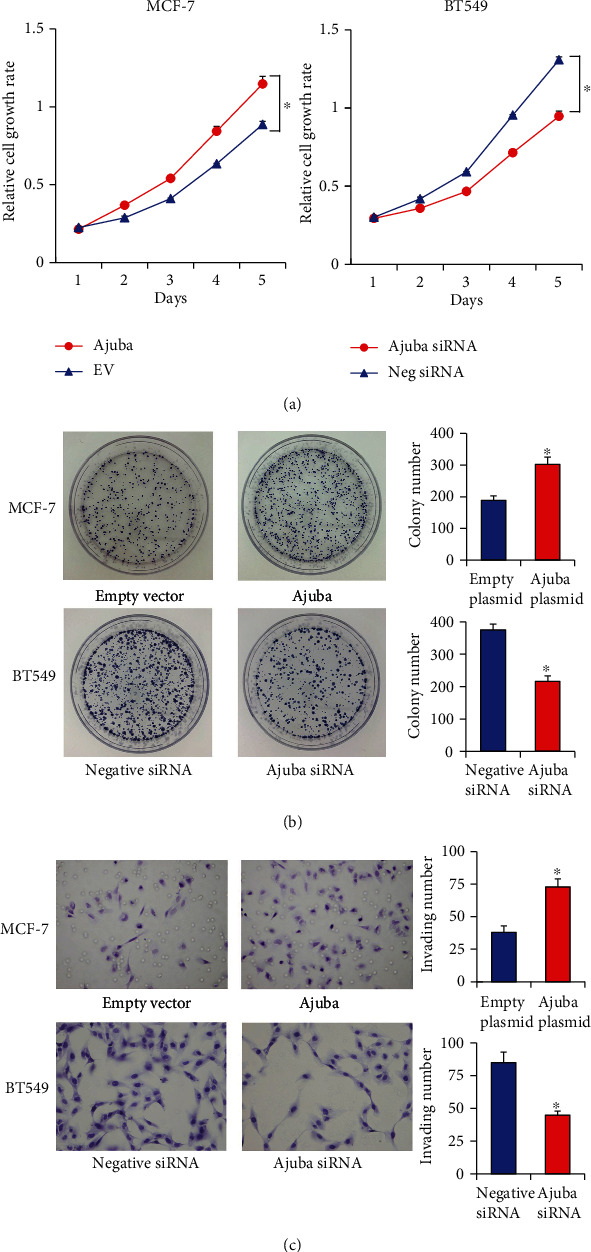
Ajuba positively regulates breast cancer cell proliferation. (a) CCK8 demonstrated that Ajuba overexpression increased the MCF-7 cell line's growth speed, while Ajuba siRNA knockdown decreased the cell growth speed in the BT549 cell line. (b) Colony formation assays demonstrated that Ajuba overexpression increased colony counts in the MCF-7 cell line. Ajuba siRNA knockdown decreased colony counts in the BT549 cell line. (c) Ajuba overexpression upregulated the invading cell number, while Ajuba knockdown downregulated the invading cell number. ^∗^*p* < 0.05.

CCK8, colony formation, and invasion assays were performed to examine the biological roles of Ajuba. CCK-8 assays demonstrated that Ajuba overexpression increased while Ajuba knockdown decreased the cell growth rate (Figure 4(a)). The colony formation assay also showed that Ajuba overexpression increased the colony number while Ajuba knockdown decreased the colony number (Figure 4(b)).

Because of the positive association between high Ajuba level and nodal status in clinical samples, we examined the change of invasion after Ajuba overexpression and knockdown. As shown in Figure 4(c), Ajuba depletion inhibited invasion while Ajuba overexpression increased invading ability in breast cancer cells.

### 3.3. Ajuba Promotes Cell Cycle Progression and Glucose Uptake in Breast Cancer Cells

**Figure 5:**
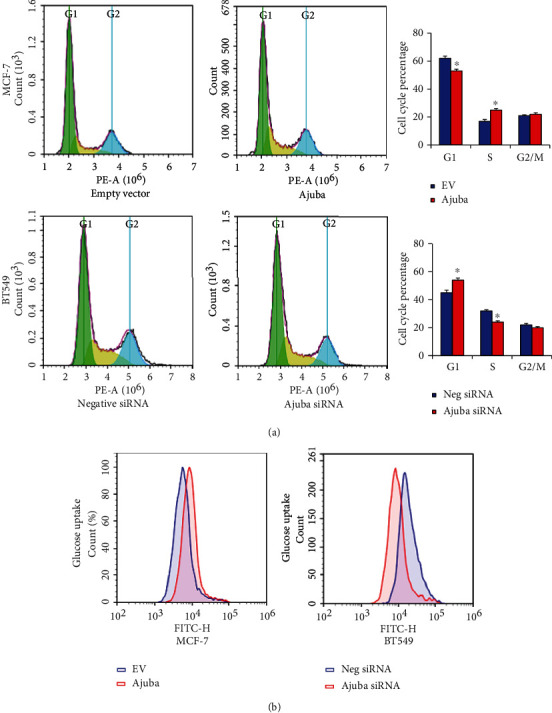
Ajuba regulates cell cycle and glucose uptake. (a) Flow cytometry demonstrated that Ajuba overexpression increased the S phase percentage in the MCF-7 cell line. Ajuba knockdown decreased the percentage of BT549 cells in the S phase. (b) 2-NBDG glucose uptake assay suggested that ectopic Ajuba expression facilitated glucose uptake rate in the MCF-7 cell line. Ajuba knockdown inhibited glucose uptake rate in BT549 cells. ^∗^*p* < 0.05.

Cell cycle analysis showed that Ajuba overexpression in MCF-7 cells increased the percentage of S phase cells and downregulated the percentage of G1 phase cells. Ajuba knockdown showed the opposite effects on BT549 cell line (Figure 5(a)). Since glucose metabolism is pivotal in the energy production of cancer cells, we examined the possible change in glucose uptake, the critical steps during glucose metabolism. The 2-NBDG glucose uptake assay demonstrated that Ajuba knockdown inhibited the glucose uptake level while Ajuba overexpression upregulated glucose uptake (Figure 5(b)).

### 3.4. Ajuba Regulates Paclitaxel Resistance in Breast Cancer Cells

**Figure 6:**
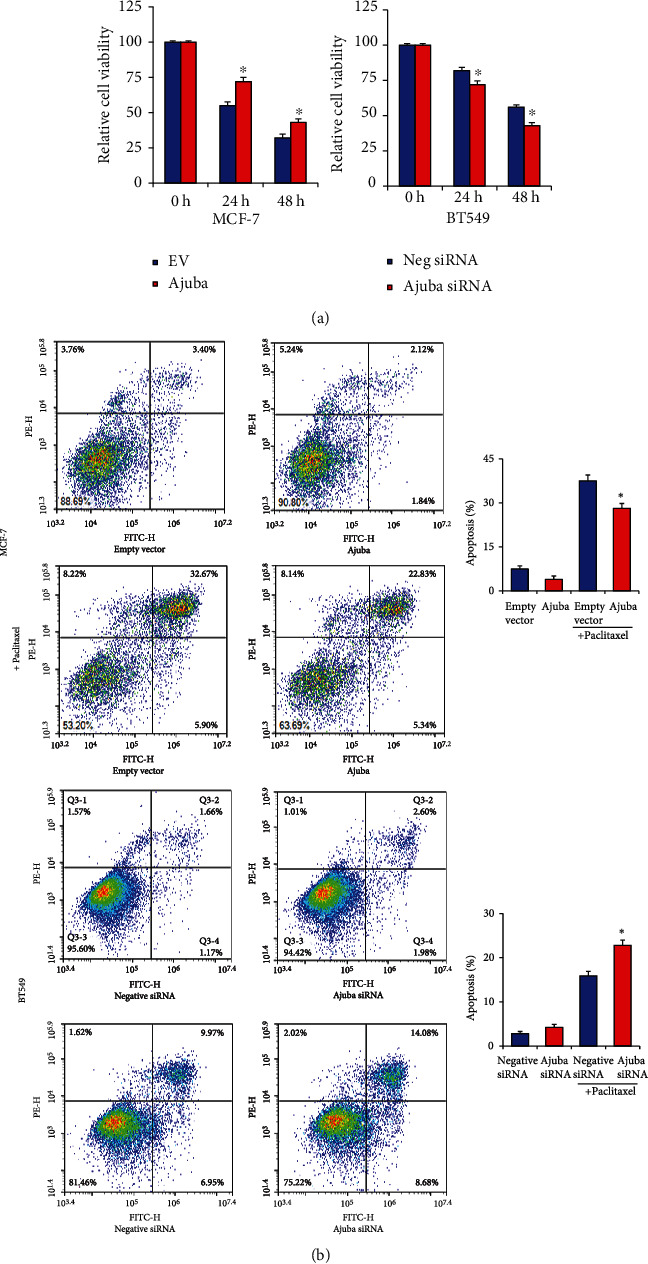
Ajuba regulates paclitaxel-induced apoptosis. (a) Breast cancer cells were treated with paclitaxel (10 nM for MCF-7; 25 nM for BT549). CCK-8 assays showed that Ajuba overexpression decreased the paclitaxel inhibition rate while Ajuba depletion increased paclitaxel inhibition. (b) Annexin V/PI analysis demonstrated that Ajuba overexpression decreased apoptosis induced by paclitaxel. Ajuba knockdown upregulated paclitaxel-induced apoptosis in BT549. Ajuba slightly reduced apoptosis in cells without paclitaxel treatment. ^∗^*p* < 0.05.

Next, we explored the role of Ajuba in chemosensitivity. MCF-7 and BT549 cells were treated with paclitaxel (10 nM for MCF-7; 25 nM for BT549, 24 hours). CCK-8 assays indicated that Ajuba knockdown upregulated the level of inhibition induced by paclitaxel while Ajuba overexpression showed the opposite effect (Figure 6(a)).

Annexin V/PI staining indicated that Ajuba knockdown upregulated the percentage of paclitaxel-induced apoptosis while Ajuba overexpression decreased the level of apoptosis, suggesting that Ajuba could induce resistance to chemotherapeutic drugs (Figure 6(b)). Ajuba could also slightly reduce apoptosis in BC cells without paclitaxel treatment.

### 3.5. Ajuba Positively Regulates TAZ-GLUT3/Survivin Signaling

**Figure 7:**
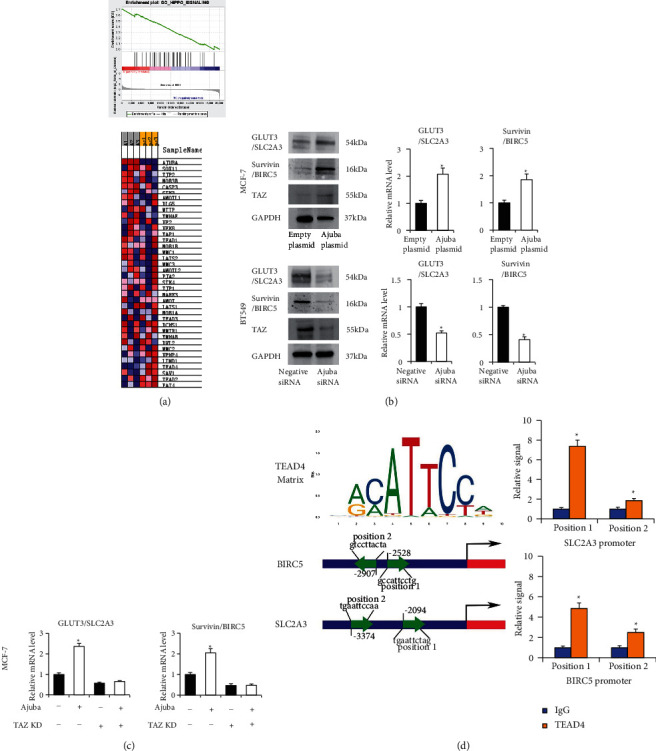
Ajuba regulates GLUT3/Survivin through TAZ. (a) RNA-sequencing and gene set expression analysis (GSEA) revealed enrichment for Hippo signaling-related genes in MCF-7 cells with Ajuba overexpression. (b) Western blotting demonstrated that Ajuba overexpression increased the protein levels of GLUT3, Survivin, and TAZ. Ajuba knockdown decreased the protein levels of GLUT3, Survivin, and TAZ. RT-qPCR showed that Ajuba positively regulated GLUT3 and Survivin mRNA in breast cancer cells. (c) TAZ siRNA was cotransfected with Ajuba plasmid in BC cells. TAZ knockdown significantly decreased the level of GLUT3 and Survivin. In TAZ depleted cells, the effects of Ajuba transfection on GLUT3/Survivin were largely abolished. (d) Predicting TEAD4 binding site and matrix using JASPAR database. Chromatin immunoprecipitation (ChIP) assay demonstrated that TEAD4 could bind to the GLUT3 and Survivin promoter regions. ^∗^*p* < 0.05.

To elucidate the potential mechanisms of Ajuba in BC, RNA-sequencing was performed to profile the global mRNA change induced by Ajuba. GSEA revealed enrichment for Hippo signaling-related genes (Figure 7(a)). We also screened several potential proteins related to glucose metabolism and chemosensitivity using western blotting. Our results showed that Ajuba increased GLUT3 and Survivin expression at both mRNA and protein levels (Figure 7(b)). Moreover, we found that Ajuba enhanced TAZ protein expression in MCF-7 cells. Ajuba depletion downregulated TAZ protein in BT549 cells (Figure 7(b)).

TAZ, the Hippo pathway's core component, has been reported to be a transcriptional regulator of many cancer-related genes. Analysis of the published Chip-Seq dataset indicated that both GLUT3/SLC2A3 and Survivin/BIRC5 are downstream targets of the Hippo signaling pathway. To further confirm their association, we used TAZ siRNA in MCF7 cells cotransfected with the Ajuba plasmid. RT-qPCR showed that TAZ knockdown significantly suppressed mRNA expression of GLUT3 and Survivin. TAZ depletion also ameliorated the effect of Ajuba overexpression on GLUT3/Survivin (Figure 7(c)).

TAZ has been reported as a transcription coactivator of TEAD4, which can bind promoter regions of Hippo target genes through the TEA domain. We examined if TEAD4 regulated GLUT3 through its promoter. JASPAR was used to predict the potential binding sites and weight matrix (Figure 7(d)). The chromatin immunoprecipitation assay (ChIP) demonstrated that TEAD4 could interact with GLUT3 and Survivin promoter regions (Figure 7(d)). The above result indicated that Ajuba could regulate GLUT3/Survivin through TAZ in BC.

### 3.6. Ajuba Promotes Tumor Growth In Vivo

**Figure 8:**
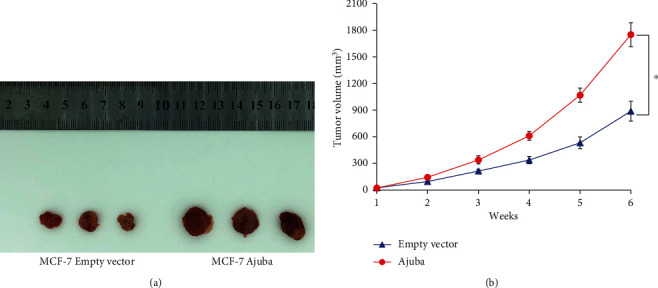
Ajuba promotes cancer cell growth in vivo. (a) MCF-7 were stably transfected with Ajuba plasmid. Cells were injected into nude mice. Representative images of tumors were shown. (b) Growth curves of Ajuba overexpressing and control MCF-7 cells. ^∗^*p* < 0.05.

To examine the effect of Ajuba on tumor growth in vivo, we established the empty vector/Ajuba overexpressing MCF-7 cell line by G418 selection. These cells were injected into nude mice subcutaneously. As shown in Figure 8, the in vivo growth rate and tumor sizes of Ajuba overexpressing MCF-7 cells were much larger than that of control cells.

## 4. Discussion

It has been recently reported that Ajuba played critical roles in a variety of human tumors. However, the involvement of Ajuba in breast cancer remains unclear. Here, we investigated the expression and tumorigenic function of Ajuba in breast cancer. Ajuba overexpression was found in 51/93 human breast cancer specimens and positively associated with TNM stage, lymph node metastasis, and poor prognosis. Interestingly, Ajuba expression was significantly enhanced in triple-negative breast cancer specimens and cell lines. The results demonstrated that Ajuba could be a promising biomarker for diagnosing aggressive breast cancer and a potential therapeutic target.

The LIM protein Ajuba acts as a scaffold participating in a diverse array of cellular processes, including cell adhesion [29, 30], mitosis [31], and apoptosis [17]. Involvement of Ajuba was reported in the oncogenic processes. We confirmed that ectopically expressed Ajuba in MCF-7 cells stimulated in vivo and in vitro proliferation, invasion, cell cycle progression, and reduced apoptosis. Our data suggest that Ajuba plays an important role in the malignant biological behavior of BC.

Our results further revealed that increased Ajuba upregulated glucose uptake, making it a positive regulator of glucose metabolism. By the fact that Ajuba increased glucose uptake, our data further demonstrated that Ajuba upregulated GLUT3 expression. GLUT family proteins, which mediate glucose transport across membranes, were reported to be elevated in human cancers [19, 32]. It has been reported that GLUT3 mediated the growth and survival of breast cancer cells [33, 34]. Survivin/BIRC5 is a member of the inhibitor of apoptosis (IAP) family. Survivin localizes in the mitochondria and inhibits apoptosis. Reports indicate that Survivin protects cancer cells from drug-induced cell death [35]. Many studies indicated that aberrant expression of Survivin is associated with poor prognosis and drug/radiation resistance in breast cancers [36–38]. Strategies targeting survivin to treat breast cancer have got promising initial results [39]. Thus, Ajuba might regulate proliferation and chemosensitivity through GLUT3 and Survivin. We also found that Ajuba upregulated TAZ protein expression. It has been reported that Ajuba could sequester the Hippo kinase complex and sustain cell proliferation by limiting YAP inhibition [14]. We also checked YAP protein levels in the current study but did not found significant change after Ajuba transfection or knockdown. TAZ has been reported to control genes regulating glucose metabolism and apoptosis [40]. TAZ is a transcription coactivator which does not directly control gene transcription. Instead, it interacts with transcription factor TEAD3/4, which directly interacts with the promoter region of target genes [41]. TAZ forms a complex with TEAD4 transcription factor to activate downstream gene transcription [42]. Using TAZ siRNA, we showed that TAZ mediated the upregulating effect of Ajuba on GLUT3/Survivin, which was also supported by ChIP results showing that TEAD4 could bind to the GLUT3/Survivin promoter region. Together, our results demonstrated a strong link among Ajuba, TAZ, GLUT3, and Survivin.

Taken together, the current study indicated that Ajuba overexpression promoted proliferation, invasion, chemoresistance, and glucose uptake in breast cancer cells and correlated with poor patient prognosis. The present study also linked its oncogenic role of Ajuba with TAZ-GLUT3/Survivin signaling, indicating the therapeutic possibility by targeting Ajuba in BC.

## Figures and Tables

**Table 1:** Distribution of Ajuba status in breast cancer according to clinicopathological characteristics.

Characteristics	Number of patients	Ajuba negative/low expression	Ajuba overexpression	*p*
Age				
<60	73	35	38	0.3027
≥60	20	7	13	
TNM stage				
I	34	23	11	0.0009
II-IV	59	19	40	
Tumor size				
<2 cm	31	18	13	0.0771
≥2 cm	62	24	38	
Lymph node metastasis				
Absent	44	28	16	0.0007
Present	49	14	35	
Estrogen receptor				
Absent	30	12	18	0.4901
Present	63	30	33	
Progesterone receptor				
Absent	43	16	27	0.1530
Present	50	26	24	
ErbB-2				
Absent	66	26	40	0.0806
Present	27	16	11	
Triple-negative				
Absent	77	39	38	0.0196
Present	16	3	13	

## Data Availability

The data that support the findings of this study are available on request from the corresponding author.
